# Effects of C-Factor on Bond Strength of Universal Adhesives to Floor and Wall Dentin in Class-I Composite Restorations

**DOI:** 10.3290/j.jad.b2701599

**Published:** 2022-03-01

**Authors:** Nafiseh Fazelian, Shahin Kasraei, Zahra Khamverdi

**Affiliations:** a Assistant Professor, Department of Restorative Dentistry, Dental School, Bushehr University of Medical Sciences, Bushehr, Iran. Wrote the manuscript, performed microscope analysis, prepared the specimens, performed the µTBS test.; b Professor, Department of Restorative Dentistry, Dental School, Shahid Beheshti University of Medical Sciences, Tehran, Iran. Developed study concept.; c Professor, Department of Restorative Dentistry, Dental School, Hamadan University of Medical Sciences, Hamadan, Iran. Performed the statistical analysis.

**Keywords:** C-factor, microtensile bond strength, universal adhesive

## Abstract

**Purpose:**

To evaluate the effects of C-factor on the bond strength of universal adhesives to floor and wall dentin in class-I composite restorations using a bulk-fill composite.

**Materials and Methods:**

108 non-carious humans third molars were randomly divided into four groups as follows: flat wall, flat floor, cavity wall, and cavity floor (n = 36). Then, each group was subdivided into three subgroups according to the type of adhesive used: Single Bond Universal, G-premio Bond (both universal adhesives), or Adper Single Bond 2 (an etch-and-rinse adhesive). After the bonding procedure, X-tra fill resin composite was applied in bulk to build up the flat surfaces or fill the cavities.Then the teeth were sectioned into 1-mm^2^ sticks and microtensile bond strength (µTBS) was measured using a universal testing machine. µTBS (MPa) was analyzed by one-way, two-way, and three-way ANOVA using SPSS Version 23 (α = 0.05).

**Results:**

Interactions between adhesives and bonding surfaces, as well as C-factor and bonding surfaces showed statistically significant differences, but the interaction between the C-factor and type of adhesive was not statistically significant. The comparison of bonded surfaces including the flat wall and the flat floor in Adper Single Bond 2 was statistically significant (p < 0.05), except for the cavity wall and cavity floor.

**Conclusion:**

Regardless of the type of adhesives, the C-factor reduced the µTBS of the composite resin to dentin. Adper Single Bond 2 mediated higher µTBS than did the universal adhesives G-premio Bond and Single Bond Universal.

Today, placing direct composite restorations is one of the most common treatments in dental practice.^6,15^ During curing of composite resins, polymerization shrinkage occurs and generates stress.^14^

The C-factor is an important clinical parameter and is related to polymerization shrinkage, bond strength, and durability.^10^ It is defined as the ratio of bonded to the unbonded surface area in the restoration. High C-factors indicate situations in which the material polymerizes under greater external constraint.^7^ In general, an increasing rate of shrinkage stress development with an increasing C-factor leads to a decreased flow capacity.^1^

Bulk-fill composites are characterized by changed filler contents or an organic matrix. It has been claimed that these composites have low polymerization shrinkage and present important advantages, eg, reducing microleakage, postoperative sensitivity, and secondary caries.^11^ These composites have specific properties, including enhanced flowability to achieve adequate adaptation to cavity walls and a minimum depth of cure of about 4 mm.^18^

The adhesion process of direct composite resin depends on several parameters, such as type of substrate (regional differences and tubular orientation of dentin), type of adhesive, ambient moisture, and the operator’s ability to perform the bonding method.^12^ One of the most recent developments in adhesive dentistry is the ‘universal’ or ‘multi-mode’ adhesives, which may be applied either in etch-and-rinse or self-etching mode, according to clinical need. These materials are simplified adhesives, usually containing all bonding components in a single bottle. Some universal adhesives may contain silane, which eliminates the silanization step in bonding to glass-ceramics or resin composites.^3^

Different types of prepared cavities have different C-factors, depending on the area of bonded surfaces, but few studies have examined the effect of C-factor on the bond strength of universal adhesives in class-I composite restorations. Thus, the aim of this study was to evaluate the effect of C-factor on the bond strength of universal dental adhesives to the dentin of the cavity floor and cavity wall in class-I composite restorations using bulk-fill composites. The hypothesis of this study was that the C-factor reduces the bond strength of universal dental adhesives in class-I composite restorations.

## Materials and Methods

### Sample Preparation

The materials, components, manufacturers and bonding procedures used in this study are presented in Table 1.

**Table 1:** Study materials

Material	pH	Composition	Application
G-Premio Bond (GPB) GC; Tokyo, Japan	1.5	10-MDP, phosphoric acid ester monomer, dimethacrylate, 4-MET, MEPS, acetone, silicon dioxide, initiators	Apply using a microbrush Leave undisturbed for 10 s after application Dry thoroughly for 5 s with oil-free air under maximum air pressure Light cure for 10 s
Single Bond Universal 3M Oral Care; St Paul MN, USA	2.7	MDP phosphate monomer, dimethacrylate resins, HEMA, Vitrebond Copolymer, filler, ethanol, water, initiators, silane	Apply the adhesive on the surface and rub it in for 20 s Gently air dry the adhesive for approximately 5 s for the solvent to evaporate Light cure for 10 s
Adper Single Bond 2 3M Oral Care	0.6	Etchant: 35% phosphoric acid (Scotchbond Etchant) Adhesive: bis-GMA, HEMA, dimethacrylates, ethanol, water, photoinitiator, methacrylate functional copolymer of polyacrylic and poly(itaconic) acids, 5-nm-diameter spherical silica particles (10% by weight)	Apply etchant for 15 s Rinse for 10 s Blot excess water Apply 2–3 consecutive coats of adhesive for 15 s with gentle agitation Gently air dry for 5 s Light polymerize for 10 s at 1200 mW/cm^2^
X-tra fil (bulk-fill) Microhybrid U VOCO Cuxhaven, Germany		Resin matrix: bis-GMA, UDMA, TEG-DMA Filler type: barium-boron-alumino-silicate glass (2–3 mm) Filler: 86 wt%	Maximum depth :4 mm 10-s curing at >1000 mW/cm^2^ Viscosity: regular

Bis-GMA: bisphenol glycidyl methacrylate; HEMA: 2-hydroxyethyl methacrylate; MDP: methacryloyloxydecyl dihydrogen phosphate; 4-MET: 4 methacryloxyethyltrimellitate anhydride;MEPS: methacryloyloxyalkyl thiophosphate.

108 intact, erupted, non-carious third molars that had been extracted (in accordance with the ethical standards of the Vice Chancellor of Research, Hamadan University of Medical Sciences) within the three last months for various reasons were collected, cleaned, and stored in 10% formalin solution. The study was approved by the ethics committee of the Vice Chancellor of Research, Hamadan University of Medical Sciences (IR.UMSHA.REC.1396.213). The teeth were mounted in auto-polymerizing acrylic resin (Acropars; Tehran, Iran) and then stored in distilled water at 24°C for 24 h before use.^2^ The teeth were randomly divided to four groups including flat wall, flat floor, cavity wall, and cavity floor.

#### Group 1: flat wall (FW)

Thirty-six teeth were sectioned along the longitudinal axis within 3 mm of the outer surface of the tooth using diamond flat fissure burs (111.534.012, Drendel and Zweiling Diamant; Berlin, Germany) in a high-speed hand piece with water coolant to expose axial-wall dentin. The cutting surface was considered as the surface of the specimen. After applying the bonding agent and composite (dimensions: 3 mm width x 5 mm length x 2 mm height), the samples were placed in a cutting machine and 1 stick was obtained from each sample.

#### Group 2: flat floor (FF)

The occlusal enamel of 36 teeth was ground away using an orthodontic trimmer (Pars Medical; Tehran, Iran) under running water, perpendicular to the longitudinal axis of the tooth, to expose a flat dentin surface. Then, 2 mm of the exposed occlusal dentin were sectioned perpendicular to the longitudinal axis using diamond flat fissure burs (see above) in a high-speed handpiece with water coolant to expose dentin. After applying the bonding agent and composite (dimensions: 3 mm width x 5 mm length x 2 mm height), the specimens were placed in a cutting machine and 1 stick was obtained from each specimen.

#### Group 3: cavity wall (CW)

**Fig 1:**
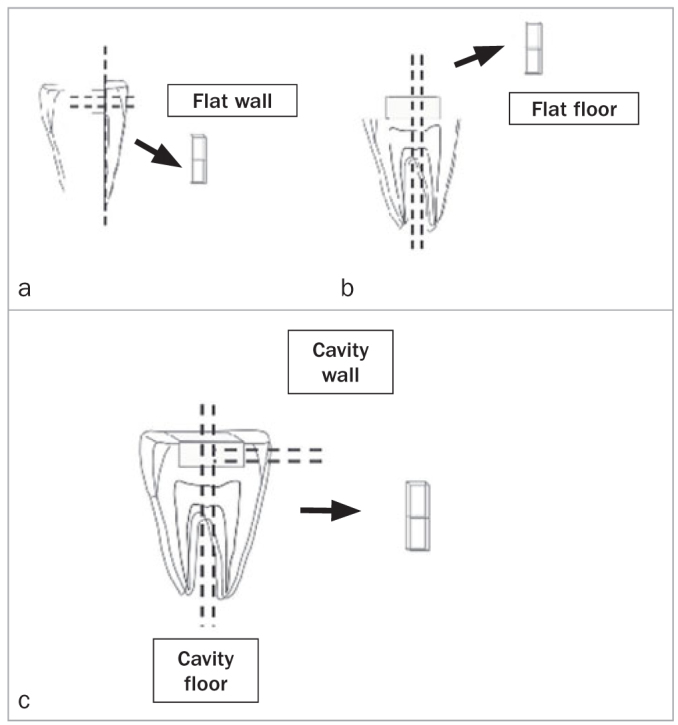
Sample preparation to microtensile bond strength test. In FF and CF groups, teeth were sectioned in two directions of mesiodistal and buccolingual and in FW and CW groups, sectioned in two directions of occlusogingival and buccolingual.

Thirty-six 36 teeth were prepared as follows: the dentin was exposed on the occlusal surface using an orthodontic trimmer (Pars Medical), class-I preparations (box-form, 3 mm width x 5 mm length x 2 mm height) within 3 mm distance of the outer axial surface of the tooth were made on the flat dentin surfaces using diamond flat fissure burs (see abov) in a high-speed handpiece with water coolant. After filling the cavity, teeth were sectioned as shown in Fig 1. One stick was obtained from each tooth.

#### Group 4: cavity floor (CF)

Thirty-six teeth were prepared as in Group 3 (Fig 1).

#### Subgroups

Finally, each of these groups was subdivided into three subgroups based on the type of adhesive used (N = 12):

- Subgroup 1 was restored with Single Bond Universal (3M Oral Care; St Paul, MN, USA) in self-etch mode, and the adhesive was light cured at 1200 mW/cm^2^ for 10 s using an LED curing unit (Kerr; Orange, CA, USA) (n = 12).
- Subgroup 2 was restored with G-premio Bond (GC) in self-etch mode, and the adhesive was light cured for 10 s as in the previous group, (n = 12).
- Subgroup 3 was restored with Adper Single Bond 2 (3M Oral Care) in etch-and-rinse mode, and adhesive was light cured for 10 s as in the previous groups (n = 12).

An X-tra fill resin composite (universal shade, Voco; Cuxhaven, Germany) was then used in bulk to build up the flat surfaces or fill the cavities. A celluloid matrix was used in FF and FW groups (3 mm width x 5 mm length x 2 mm height) to shape the resin composite, which was then light cured at 1200 mW/cm^2^ for 40 s using the same curing unit.^4^

### Microtensile Bond Strength Test

Specimens were stored in distilled water 24 h at 37°C. The samples were prepared as follows: In groups FF and CF, teeth were sectioned in two directions – mesiodistally and buccolingually – using a diamond disk (Mashhad Nemov Company; Mashhad, Iran) in a low-speed cutting machine operating at 300 rpm under water coolant to obtain dentin-composite sticks with a cross-section of approximately 1 mm^2^. The same protocol was perfomed in groups FW and CW. Each tooth was sectioned in two directions: occlusogingivally and buccolingually (Fig 1).^23^

Each prepared stick was transferred to a universal testing machine (SANTAM, SMT20; Tehran, Iran). The samples were glued to the device using cyanoacrylate adhesive (Loctite Super Bonder Gel Control, Henkel; São Paulo, Brazil). The bonding area was positioned vertically relative to the direction of tensile loading. Tensile load was applied to the resin-dentin interface at a crosshead speed of 0.5 mm/min (ISO TR 1145) until failure. The data for each group were recorded in Newtons (N) and converted to megapascals (MPa) by dividing the load in N by the surface area in mm^2^. In order to compare the mean μTBS of the studied groups, one-way, two-way, and three-way ANOVA and Dunnett’s test were used in SPSS Version 23. p < 0.05 was considered statistically significant.

### Stereomicroscopic Analysis

To determine the fracture pattern, each sample was examined under a stereomicroscope (SZ40, Olympus; Tokyo, Japan) with a magnification of 40x. Three failure modes were determined: 1. adhesive (interfacial) fracture: fracture at adhesive- or adhesive-dentin interface; 2. cohesive fracture: fracture within dentin or composite; 3. mixed fracture: a combination of adhesive and cohesive fracture.

### Scanning Electron Microscope (SEM) Analysis

SEM analysis was used to investigate the surface morphology of the specimens. For this purpose, the selected specimen was affixed to an aluminum stub by a conductive adhesive tape (double-sided carbon tape) and were then sputter-coated (JFC-1100E Ion Sputter, JEOL; Tokyo, Japan) with gold-palladium alloy for 10 min. Specimens were observed in an SEM (JEOL JSM-840A) at magnifications of 500X and 1000X.

## Results

### Microtensile Bond Strength

Table 2 presents the µTBS of each group. During the cutting procedures, pre-test failures occurred in all groups and were assigned a value of 0 MPa. The Smirnov-Kolmogorov test confirmed that the data were normally distributed (p > 0.05). The highest and lowest mean µTBS and standard deviations were obtained using G-premio Bond (FF) (18.01 ± 9.12 MPa) and Single Bond Universal^13^ (3.63 ± 2.01 MPa), respectively.

**Table 2:** Mean microtensile bond strength of the bonding adhesives

Adhesives	Surface	Mean	Standard deviation	Max	Min
G-premio Bond	FF	18.01	9.12	42.70	9.50
	FW	15.64	6.21	26.30	5.70
	CF	5.04	1.96	8.50	2.40
	CW	5.26	1.67	8.10	1.90
Single Bond Universal	FF	12.83	7.90	25.30	3.20
	FW	15.25	3.05	19.80	13.20
	CF	4.65	2.51	10.50	1.80
	CW	3.63	2.01	7.95	1.30
Adper Single Bond 2	FF	14.22	5.58	29.01	7.03
	FW	28.92	8.75	44.10	17.80
	CF	10.70	4.35	18.10	3.60
	CW	8.74	4.42	15.80	3.10

According to the results of three-way ANOVA, µTBS was significantly affected by the three main factors “C-factor”, “bonding surfaces” and “type of adhesive”.

There was a significant difference in µTBS depending on the type of adhesive (Tables 3 and 4). However, the interaction between C-factor and type of adhesive did not produce significant differences in µTBS (p = 0.714). However, the interactions between type of adhesive x bonding surface and C-factor x bonding surface yielded statistically significant differences in µTBS (p < 0.05).

**Table 3:** Tukey’s HSD test

Adhesive	Adhesive	Mean difference	Sig.
G-premio Bond	Adper Single Bond 2	-13.28500	0.000*
	Single Bond Universal	0.39000	0.994
Single Bond Universal	Adper Single Bond 2	-13.67500	0.005

Mean difference is significant at p < 0.05.

**Table 4:** Interaction among “C-factor”, “bonding surface,” and “type of adhesive”

	df	Mean square	F	Sig.
Type of adhesives	2	479.389	15.202	0.000*
$\text{C-factor}=\frac{\mathrm{bonded surface area}}{\mathrm{unbonded surface area}}$	1	3942.184	125.014	0.000*
Bonding surfaces	1	126.530	4.012	0.047*
Adhesive x C-factor	2	10.639	.337	0.714
Adhesive x bonding surface	2	184.519	5.851	0.004*
C-factor x bonding surface	1	270.272	8.571	0.004*
Adhesive x C-factor x bonding surface	2	300.000	9.514	0.000*

* Mean difference is significant at p < 0.05.

Although the results obtained from HSD Tukey’s HSD test (adhesives) showed significant differences between Adper Single Bond 2 and the universal adhesives (G-premio Bond and Single Bond Universal), G-premio Bond and Single Bond Universal produced statistically similar results.

A comparison of the type of bonded surfaces, that is, FF vs CW and FF vs CF, yielded signifcant differences in µTBS (p < 0.05; Table 4).

The results obtained from one-way ANOVA showed that the mean μTBS values were not significantly different in all three adhesives in FF groups. Also, the mean μTBS in Adper Single Bond 2 adhesive was significantly higher than that of the universal adhesives (p = 0.05).

### Stereomicroscopic Failure Mode Analysis

Adper Single Bond 2 and Single Bond Universal had the highest proportion of adhesive fractures on FW and lowest adhesive fracture on CW.

With G-premio Bond, the highest rate of adhesive fractures was on FF and the lowest on CW and CF. This fracture pattern was consistent with its microtensile bond strength (Table 5); adhesive fractures are more frequent with higher µTBSs.

**Table 5:** Failure mode distribution

Adhesive	Surface	Adhesive	Mixed	Cohesive	
				Composite	Dentin
G-premio Bond	FF	6	5	1	0
	FW	5	6	1	0
	CF	4	5	1	2
	CW	4	4	3	1
Single Bond Universal	FF	4	5	2	1
	FW	5	4	2	1
	CF	3	2	3	4
	CW	2	3	2	5
Adper Single Bond 2	FF	5	6	1	0
	FW	8	3	1	0
	CF	5	3	1	3
	CW	4	4	2	2

### SEM Surface Morphology Analysis

With the universal adhesives, fractures occurred at the top of the hybrid layer, while with Adper Single Bond 2 (etch-and-rinse adhesive), fractures occurred at the bottom of the hybrid layer.

**Fig 2:**
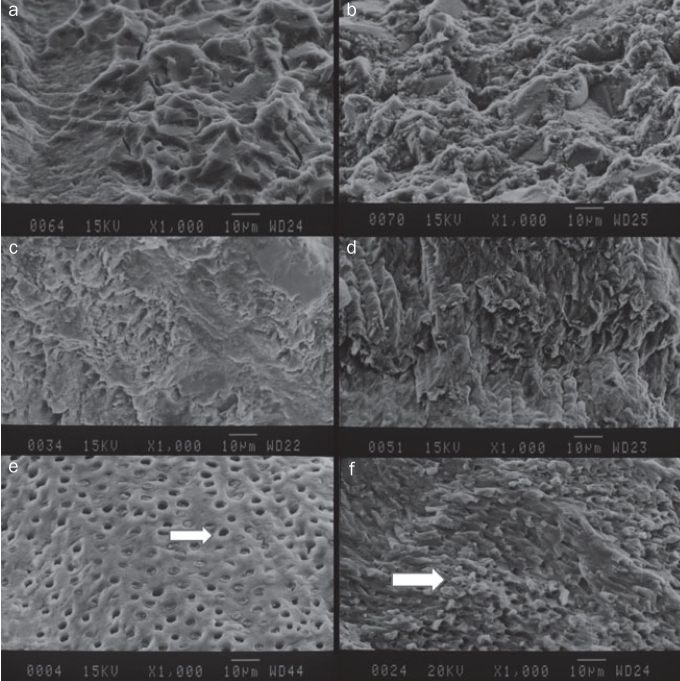
SEM micrographs. a: G-premio Bond, floor; b: G-premio Bond, wall; c: Single Bond Universal, floor; d: Single Bond Universal, wall; e: Adper Single Bond 2, floor; the tubule apertures are opened and widened by acid application (white arrow). f: Adper Single Bond 2, wall; resin tag formation (white arrow).

It was also observed that application of acid with the etch-and-rinse adhesive led to wider lumens and funnel-shaped openings, as well as resin tags in the open dentinal tubules (Fig 2).

## Discussion

In recent years, the demand for posterior resin composite restorations has increased because they are tooth colored, lack mercury, are thermally nonconductive, biocompatible, and can bond to tooth structure.^9^

However, an important drawbacks inherent in this material is the stress induced by polymerization contraction.^14^ The purpose of this study was to evaluate the effects of C-factor on the bond strength of universal adhesives at the cavity floor and cavity wall in class-I composite restorations.

Universal adhesives can be used in self-etch mode, which keeps demineralized dentin moist and prevents the collagen collapse.^8^ When a universal adhesive is used in self-etch mode, the hybrid layer contains residual hydroxyapatite and smear layer. Furthermore, self-etch adhesives are less technique sensitive and can be easily used under difficult moisture-control conditions, especially in the posterior teeth.^5^ Therefore, the present study used Single Bond Universal and G-premio Bond in self-etch mode.

In this study, the µTBS test was used, which is a valid method for measuring bond strength. This test is able to more accurately measure tensile bond strength. Furthermore, it is allows the examination the interfacial bond strength in areas smalller than 1 mm^2^.^16^

C-factor, defined as the ratio of bonded to unbonded surface area in the restoration, is higher in box-shaped cavities such as class-I preparations.^17^

According to the results of this study, C-factor statistically significantly influenced µTBS. For example, when composite resin is inserted into the cavity and light cured, the bond strength can be significantly reduced due to the polymerization shrinkage.^24^ Yoshikawa et al^24^ studied the effects of dentin depth and C-factor on bond strength using Clearfil Liner Bond 2 (Kuraray Noritake; Osaka, Japan), One-Step (Bisco; Schaumburg, IL, USA), and Super Bond D (Sun Medical; Shiga, Japan) liner and found that all groups had the highest bond strength at the unbonded surface. Also, in the presence of C-factor, the bond strength of all groups decreased.^24^

In this study, the type of adhesive had a significant effect on the μTBS. Adper Single Bond 2, an etch-and-rinse adhesive, showed higher μTBS than the two self-etching universal adhesives.

In a more recent study by Yoshikawa et al^23^ on the effects of C-factor on bond strength to floor and wall dentin, the interaction between C-factor and type of adhesive was meaningful; however, in the present study, the interaction between C-factor and adhesive type was not significant, and bond strength was unrelated to the type of adhesive. The reason for this disagreement with the present study can be attributed to the type of adhesive. Yoshikawa et al^23^ used Clearfil tri-S Bond and Clearfil SE Bond, but this study evaluated two universal adhesives and one etch-and-rinse adhesive.

In the present study, G-premio Bond, Single Bond Universal, and Adper Single Bond 2 showed similar µTBS given the same C-factor (ie, 5). This may be due to the combination of universal adhesives and the presence of 10-MDP monomer in universal adhesives, which forms a chemical bond to the tooth structure. This showed a µTBS similar to Adper Single Bond 2, which was used as the gold-standard bonding technique.^22^

On FF surfaces, G-premio Bond yielded higher µTBS, which may be attributed to the presence of 10-MDP monomer. When a universal adhesive is used in self-etch mode, the etched region is not rinsed. Thus, calcium and phosphate molecules that formed by dissolving the hydroxyapatite crystals bond chemically to the 10-MDP monomer.^22^

Using SEM and TEM, Van Meerbeek et al^20^ compared the ultrastructure of the resin-dentin interdiffusion zone of the Clearfil Liner Bond system and showed that the orientation of dentin tubules could have a significant effect on the morphology of theh hybrid layer induced by etch-and-rinse adhesives. This is in line with present study. As shown in Table 2, there was a significant interaction between the orientation of dentin tubules and the type of adhesive. The µTBS strength with application of Adper Single Bond 2 on FW was higher than on FF. In the study by Van Meerbeek et al,^20^ when the orientation of dentin tubules was perpendicular to the cavity floor, the hybrid layer thickened and the resin tags were longer. Also, when the orientation of dentin tubules was parallel or at least not perfectly perpendicular to the wall, the hybrid layer was thinner and resin tags were absent.^20^ The difference may be related to the adhesive; Clearfil Liner Bond consists of two separate bottles: primer and MDP adhesive resin, but Adper Single Bond 2 is a one-bottle adhesive and does not contain MDP monomer. Two-bottle adhesives provide adequate conditioning-priming pretreatment and diffusibility of the resin monomers.^19^

Single Bond Universal had the lowest bond strength on FF because it contains HEMA and MDP monomers. HEMA and polyalkenoic acid copolymer compete with the 10-MDP monomer to bind with the surface of hydroxyapatite crystals. It can reduce the formation of calcium 10-MDP salts in the resin-dentin interface.^21^

Although there was a significant difference between the μTBS of Adper Single Bond 2 and the universal adhesives on FW, the μTBS of these two adhesives to FF was about the same.

Overall, this study demonstrated that the C-factor reduced the bond strength of universal dental adhesives in class-I composite restorations; thus, the null hypothesis is accepted. To reduce polymerization shrinkage stress related to high C-factor cavity preparations, the restorative technique used (bulk or incremental filling, curing method, low-shrinkage restorative materials) is important.

## Conclusion

- The type of adhesive had a significant effect on the μTBS. The etch-and-rinse adhesive mediated higher μTBS than did the universal adhesives.
- To reduce polymerization shrinkage stress related to the C-factor, the type of adhesive is not important.
- There is a significant interaction between the orientation of dentin tubules and the type of adhesive used.

## Clinical relevance:

The type of dental adhesive does not affect the polymerization shrinkage stress related to C-factor. Thus, in clinical usage, restorative techniques (bulk or incremental filling, curing method, low-shrinkage restorative materials) must be considered.
